# Circ-PAN3 facilitates hepatocellular carcinoma growth via sponging miR-153 and upregulating cyclin D1

**DOI:** 10.32604/or.2024.046774

**Published:** 2025-01-16

**Authors:** SHUO YU, MIN WANG, XU LI, XINGJUN GUO, RENYI QIN

**Affiliations:** Department of Biliary-Pancreatic Surgery, Affiliated Tongji Hospital, Tongji Medical College, Huazhong University of Science and Technology, Wuhan, 430030, China

**Keywords:** Hepatocellular carcinoma, Circ-PAN3, miR-153, RNA sponge, Cyclin D1

## Abstract

**Background:**

Circular RNAs (circRNAs) play a pivotal role in the development and advancement of various cancer types. However, the involvement of circ-PAN3 in hepatocellular carcinoma (HCC) is not well understood. To shed light on this, we conducted a comprehensive study through biochemistry, cell biology, molecular biology, and bioinformatics techniques to investigate the role of circ-PAN3 and its associated pathway in the progression of HCC.

**Methods:**

Cell Counting Kit-8 (CCK-8) assay and colony formation assay were utilized to evaluate cell proliferation; Quantitative real-time PCR (RT-qPCR) and Western blot were adopted for assessing mRNA and protein expression; Annexin V/propidium iodide (PI) staining was applied to detect cellular apoptosis; CircInteractome and Targetscan databases were searched to predict potential targets of circRNA and miRNA; Luciferase reporter assay and RNA pull-down assay were performed to examine the interaction of RNA molecules.

**Conclusions:**

Our findings revealed a significant increase in circ-PAN3 expression in HCC clinical specimens, which correlated with a poor survival rate in HCC patients. Knockdown of circ-PAN3 resulted in impaired cell proliferation, reduced cell survival, and inhibited tumorigenesis of HCC *in vivo*. Further analysis demonstrated that circ-PAN3 could serve as a sponge for miR-153, leading to a decrease in its expression level. This in turn upregulated cyclin D1 and ultimately promoted the proliferation of HCC cells. Additionally, overexpression of cyclin D1 mitigated the inhibitory effect on HCC proliferation induced by circ-PAN3 knockdown. Our study highlights the presence of a novel circ-PAN3/miR-153/cyclin D1 regulatory axis that plays a crucial role in the progression of HCC.

## Introduction

Hepatocellular carcinoma (HCC) is a notorious malignancy that has a significant impact globally, resulting in approximately 1 million casualties each year [1,2]. Unfortunately, due to limited diagnostic methods, most patients are diagnosed at an advanced stage, making them ineligible for liver transplantation or surgical resection [3,4]. Additionally, HCC is characterized by its high aggressiveness, invasiveness, and frequent relapses after resection [3], all of which contribute to a poor prognosis for HCC patients [1]. Therefore, it is crucial to unveil the molecular mechanisms underlying the occurrence and progression of HCC in order to identify new therapeutic targets.

Circular RNAs (circRNAs) belong to a novel class of endogenous noncoding RNAs that are generally produced by exon scrambling during the splicing process, without a 5’ cap or a 3’ Poly A tail [5,6]. There are four types of circRNAs based on their sources: exonic circRNAs (ecircRNA), intronic circRNA, exonic-intronic circRNA (EIciRNA), and intergenic circRNAs. Among these, ecircRNA accounts for 80% of circRNAs [6]. CircRNAs are known to participate in various regulatory mechanisms, including competitive endogenous RNA binding (ceRNAs), protein interactions, and transcriptional or translational regulation [7–9]. Recent studies have shown that dysregulation of circRNAs is associated with the progression and prognosis of hepatocellular carcinoma (HCC) through different modes of actions [10–12]. For instance, while circ_0091570 has been found to suppress HCC progression by sponging miR-1307 [11] and circMTO1 inhibits HCC progression through targeting miR-9/p21 axis [13], circSLC3A2 has been reported to promote HCC progression by sponging miR-490-3p and regulating PPM1F expression [14]. Another newly identified circRNA, circ-PAN3, has been implicated in drug resistance in acute myeloid leukemia and the self-renewal of intestinal stem cells [15,16]. Given the growing evidence supporting the importance of circRNAs in HCC development, we wonder whether circ-PAN3 is also engaged in the progression of HCC.

Cyclin D1 serves as a critical cell cycle regulator which is essential for the transition from G1 to S phase during the cell cycle progression in mammalian cells [17]. Aberrant expression of cyclin D1 has been linked to the induction of oncogenic responses and has been associated with poor survival in cancer patients [18]. The transcription of cyclin D1 can be upregulated by X-linked inhibitor of apoptosis protein (XIAP) through E3 ligase-mediated phosphorylation [19]. Interestingly, it has been found that circ-PAN3 can positively regulate XIAP expression, leading to chemoresistance in acute myeloid leukemia [15]. The convergence of circ-PAN3 and cyclin D1 with XIAP, along with the upregulation of both circ-PAN3 and cyclin D1 in several malignancies, suggests that circ-PAN3 may be engaged in regulating cell cycle progression.

In the present study, our objective was to investigate the impact of circ-PAN3 on the progression of HCC and scrutinize the underlying mechanism. Our findings revealed that circ-PAN3 plays a crucial role in promoting HCC cell proliferation through the circ-PAN3/miR-153/cyclin D1 regulatory axis. These results provide significant evidence supporting the potential of circ-PAN3 as a valuable diagnostic and therapeutic target for the diagnosis and treatment of HCC.

## Materials and Methods

### Ethics, patients and human tissues

The protocol of this study involving the usage of human tissues was reviewed and approved by the Ethics Committee of Medical Research at the Affiliated Tongji Hospital, Tongji Medical College, Huazhong University of Science and Technology (2019-045). Patients were required to provide informed consent for the use of tumor and adjacent tissues at the same hospital.

A total of 80 pairs of HCC tumor tissues and adjacent normal tissues were collected at Affiliated Tongji Hospital, Tongji Medical College, Huazhong University of Science and Technology between January 2010 and December 2015. The tumor-adjacent tissues were obtained at a distance of at least 1 cm from the tumor tissues. Pathological analysis confirmed the absence of cancer cells in the tumor-adjacent tissues, which was independently confirmed by two pathologists. R0 resection was performed for all the patients involved in this study. None of the patients had received chemotherapy, radiotherapy, interventional therapy, or targeted therapy prior to surgery. Patients with 2 or more malignancies or those who died due to HCC within 1 month after surgery were excluded from the study. The overall survival (OS) period was defined as the time interval between the date of surgery and either the end of follow-up or the date of death.

### Cell line culture

HCC cell lines, including Huh7, HCCLM3 and SK-HEP-1, as well as the human normal liver cell line THLE-3 were purchased from Cell Bank of the Chinese Academy of Sciences (Chinese Academy of Sciences, Shanghai, China). Cells were cultured in DMEM (11965092, Invitrogen, Shanghai, China) containing 10% fetal bovine serum (A5670701, Invitrogen, Shanghai, China), penicillin (15140122, 100 U/mL; Invitrogen, Shanghai, China), and streptomycin (15140122, 100 U/mL; Invitrogen, Shanghai, China). All cells were cultivated in a humidified incubator (Thermo Fisher Scientific, Inc., San Diego, CA, USA) at 37°C with 5% CO_2_. All cell lines have been tested to eliminate mycoplasma contamination.

### CircRNA microarray

The expression of circRNAs in seven pairs of HCC tissues and adjacent tissues was profiled through circRNA microarray analysis (KangChen Biotech, Shanghai, China). Briefly, total RNA sample was amplified and transcribed into fluorescent cRNA using random primers. Labeled cRNAs were then hybridized onto an Arraystar Human circRNA Array Chip (AS-S-CR-H-V2.0, Arraystar Inc., Rockville, MD, USA). Sample preparation and microarray hybridization followed the manufacturer’s protocols. The fluorescence signal of hybridization was scanned using an Axon GenePix 4000B microarray scanner (Molecular Devices, Sunnyvale, CA, USA), and the scanned images were imported into GenePix Pro 6.0 software (Molecular Devices, Sunnyvale, CA, USA) for grid alignment and data extraction. Quantile normalization and subsequent data processing were performed using R studio software (version 4.2.0, RStudio, Inc., Boston, MA, USA).

### Bioinformatic analysis

The prediction of circRNA targets and miRNA targets was conducted using the following online resources: CircInteractome (https://circinteractome.nia.nih.gov/), miRanda (http://starbase.sysu.edu.cn), Diana-tools (http://diana.imis.athena-innovation.gr/), TargetScan (https://www.targetscan.org/), miRanda (http://www.miranda.org.uk/), miRBridge (http://mirbridge.org/), and Pictar (http://mirsystem.cgm.ntu.edu.tw/index.php).

### Cell transfection

HCC cells were inoculated into six-well plates and grown to 60%–70% confluency prior to the transfection. Small interfering RNAs (siRNAs) were designed and synthesized from MedChemExpress (Shanghai, China) A total of 100 nM si-circ-PAN3#1, si-circ-PAN3#2 or si-NC was introduced into HCC cells using Lipofectamine™ 3000 (L3000008, Invitrogen, Shanghai, China) at 37°C for 48 h, according to the manufacturer’s protocols. The transfection doses of miR-153 mimic or inhibitor and their corresponding controls (RiboBio, Guangzhou, China) were 100 nM for cells in each well of 6-well plates. To overexpress cyclin D1, cyclin D1 expression plasmid (pReceiver-M02-cyclin D1) and the empty vector (pReceiver-M02) were produced by Genscript (Nanjing, China). Plasmid was transfected at 6 μg/well using Lipofectamine™ 3000. The sequences of the transfected mimic, inhibitor and siRNA were as follows: si-circ-PAN3#1: 5′-UCUGACCCAAAACAACCCCdTdT-3′; si-circ-PAN3#2: 5′-GAGAAAGUGGGGAAUGUCGUUdTdT-3′; si-NC: 5′-UUCUCCGAACGUGUCACGU-3′; miR-153 mimic: 5′-UUGCAUAGUCACAAAAGUGAUC-3′; NC-mimic: 5′-ATCGTGCTAGTCGATGCTAGCT-3′; miR-153 inhibitor: 5′-GAUCACUUUUGUGACUAUGCAA-3′; NC-inhibitor: 5′-CGATCGCAGCGGTGCAGTGCG-3′.

### RNA extraction and reverse transcription-quantitative PCR (RT-qPCR)

RNA samples from cultured cell lines and clinical specimens were isolated using TRIzol® reagent (15596018CN, Invitrogen, Shanghai, China). Subsequently, the PrimeScript RT master mix (RR036A; Takara Bio, Inc, Dalian, China) was employed to convert RNA sample (1000 ng) into cDNA. Quantitative PCR analysis of cDNA was performed using the SYBR Premix Ex Taq II kit (DRR081A; Takara Bio, Inc., Dalian, China) on the LightCycler 96 qPCR system (Roche Diagnostics, Basel, Switzerland). The thermocycling conditions were as follows: initial denaturation at 95°C for 30 s; 40 cycles of 5 s at 95°C, 1 min at 60°C, and 15 s at 72°C; with a final extension cycle at 72°C for 5 min. The expression levels of circRNAs and mRNAs were normalized to the expression of glyceraldehyde-3-phosphate dehydrogenase (GAPDH) mRNA. For miRNA expression analysis, One Step PrimeScript miRNA cDNA Synthesis Kit (D1801, Haigene, Hangzhou, China) was used for cDNA synthesis, and U6 snRNA was utilized as a normalized control. The sequences of primers used for qPCR were as follows: circ-PAN3: Forward: GATTTCCATGCTGGAGGAGA, Reverse: GGAATGAAGAGGGGAAGACC; miR-153: Forward: TTGCATAGTCACAAAAGTGAT, Reverse: CAGTGCGTGTCGTGGAGT; Cyclin D1: Forward: GCTGCGAAGTGGAAACCATC, Reverse: CCTCCTTCTGCACACATTTGAA; GAPDH: Forward: CGGAGTCAACGGATTTGGTCGTAT, Reverse: AGCCTTCTCCATGGTGGTGAAGAC; U6 Forward: CTCGCTTCGGCAGCACA, Reverse: AACGCTTCACGAATTTGCGT. The relative levels were calculated using the 2^−ΔΔCq^ method [20].

### Cell proliferation assay

Cell proliferation was assessed using the Cell Counting Kit-8 (CCK-8) assay kit (CK04, Dojindo Molecular Technologies, Inc. Rockville, MD, USA). Following transfection, cells were seeded into 96-well plates at a density of 2 × 10^3^ cells/well. The cells were then cultured at 37°C for 24, 48, and 72 h. After the respective incubation periods, 10 μL of CCK-8 solution was added to the culture medium in each well, followed by 90-min incubation at 37°C. The absorbance was measured at 450 nm using a spectrophotometer (BioTek Instruments, Inc., Winooski, VT, USA). The relative cell viability was calculated as follows: (absorbance at 450 nm of the treated group—absorbance at 450 nm of the blank)/(absorbance at 450 nm of the control group—absorbance at 450 nm of the blank) × 100%.

### Colony formation assay

Transfected cells were plated into 6-well plates at a density of 2000 cells/well and incubated for 14 days at 37°C. After that, the cells were washed with PBS and fixed using 4% paraformaldehyde. Subsequently, the cells were stained with 0.1% crystal violet (V5265, Sigma-Aldrich; Merck KGaA, Darmstadt, Germany) for 20 min at room temperature. The stained colonies were observed and counted under a Leica microscope with AMD processor (DM500, Leica, Bensheim, Germany).

### Apoptosis analysis

To detect apoptotic events, transfected cells were seeded and maintained for 48 h. After that, the cells were collected, washed twice with PBS, and re-suspended in the Annexin-V staining buffer. Staining of apoptosis was performed using an annexin V-fluorescein isothiocyanate apoptosis detection kit (556547; BD Biosciences, San Diego, CA, USA) following the manufacturer’s manual. Subsequently, cell apoptosis frequency was quantified on the BD FACSVantage™ SE flow cytometer (BD Biosciences, San Diego, CA, USA), and data analysis was conducted using Flow Jo 7.6 software (FlowJo LLC, Ashland, OR, USA).

### Cell cycle analysis

For cell cycle analysis, cells were harvested by trypsin digestion and then fixed in 70% ethanol at 4°C overnight. Afterward, cells were centrifugated for 5 min at 1600 × g at 4°C, and DNA contents were stained using the Cycle TEST DNA Reagent kit (340242; BD Biosciences, San Diego, CA, USA). Cell sample was processed on the BD FACSVantage™ SE System (BD Biosciences, San Diego, CA, USA), and data analysis was conducted using Flow Jo 7.6 software (FlowJo LLC, Ashland, OR, USA).

### Luciferase reporter assay

Cells were cultured until 60%–70% confluence prior to transfection. circ-PAN3-WT vector or circ-PAN3-mutant (MUT) sequences were cloned into pmirGLO reporter vector (Promega Corporation, Madison, WI, USA), and CCND1-WT and CCND1-MUT luciferase reporter vector were constructed similarly. Cells were co-transfected with WT or MUT reporter and miRNA mimic or NC mimic using Lipofectamine 3000. After the transfection for 48 h, luciferase activity was detected using a Dual-Luciferase Reporter Assay System (E1910; Promega Corporation, Madison, WI, USA), with the firefly luciferase activity being normalized to Renilla luciferase activity.

### RNA pull-down assay

HCC cells lysed using pull-down lysis buffer from the EZ-RIP assay kit (17-701, Sigma, Darmstadt, Germany) on ice for 15 min. Afterward, 1 mL cell lysate was incubated with 100 nM Biotin-circ-PAN3 probe, Biotin-cyclin D1 mRNA probe, or the biotin-control probe for 4 h on ice, followed by another 2-h incubation with 100 μL C-1 magnetic beads (65001, Life Technologies, Waltham, MA, USA). After washing with the lysis buffer for 4 times, the RNA sample bound to the beads was eluted and extracted with a RNeasy Mini Kit (74104, QIAGEN, Hilden, Germany) for subsequent RT-qPCR.

### Protein extraction and western blot analysis

Proteins from cells and tissues were isolated using RIPA buffer (P0013B; Beyotime Biotechnology, Beijing, China) supplemented with an EDTA-free protease inhibitor cocktail (04693159001; Roche Diagnostics, Basel, Switzerland) on ice for 15 min. The protein concentration was determined using a BCA assay kit (23227; Thermo Fisher Scientific, Inc. Waltham, MA, USA). The extracted proteins were separated using 10% sodium dodecyl sulfate-polyacrylamide gel electrophoresis, and then trans-blotted to polyvinylidene difluoride (PVDF) membranes (03010040001, Sigma, Darmstadt, Germany). Then the membranes were blocked with 5% skimmed milk in TBS with 0.05% Tween-20 for 1 h at room temperature. Subsequently, the membranes were incubated with primary antibodies at a 1:1000 dilution at 4°C for 18 h. Next, the membranes were incubated with horseradish peroxidase-labeled secondary antibodies (ab181658; Abcam, Cambridge, UK) at a 1:5000 dilution for 1 h at room temperature. An enhanced chemiluminescence kit (407207; EMD Millipore, Darmstadt, Germany) was used to visualize the target proteins on the Tanon 4600 imaging system (Tanon Science and Technology Co., Ltd., Shanghai, China). The primary antibodies were Cyclin D1 (cat. no. 55506S; Cell Signaling Technology, Inc., Danvers, MA, USA), and GAPDH (no. ab128915; Abcam, Cambridge, UK).

### Xenograft tumor formation model of HCC cells

Experiments involving animals were reviewed and approved by the Animal Welfare Committee of Affiliated Tongji Hospital, Tongji Medical College, Huazhong University of Science and Technology (2022DL033). Male BALB/c nude mice (4 weeks old; weight, 18–20 g; 6 animal per group) were purchased from the Model Animal Research Center of Nanjing University (Nanjing, China). Huh7 cells (3 × 10^6^) in serum-free DMEM medium were injected into the nude mice’s right flank. The mice were administrated with si-NC or si-circPAN3 at 0.5 mg/kg/week using EntransterTM *in vivo* RNA transfection reagent (18668-11-1, Engreen Biosystem Co., Ltd., Beijing, China) via intraperitoneal injection. The tumor volume (V) was monitored every three days and calculated using the following formula: V = length × width^2^/2. After 4 weeks, all the mice were sacrificed via cervical dislocation and the xenograft tumor tissues were harvested for further analysis.

### Statistical analysis

Student’s *t*-test, χ^2^ test, or one-way ANOVA were implemented using the GraphPad Prism v8.0 (GraphPad Software, Inc., La Jolla, CA, USA). Survival curve was plotted based on Kaplan-Meier method, and a log-rank test was used for statistical comparison. The correlation between the expression levels of two molecules was analyzed using Pearson’s correlation analysis. *p* < 0.05 was considered to be statistically different. Data were displayed as mean ± Standard Error of the Mean (SEM) from three independent experiments.

## Results

### Circ-PAN3 is overexpressed in HCC tissues and its high expression is associated with poor prognosis

**Figure 1:**
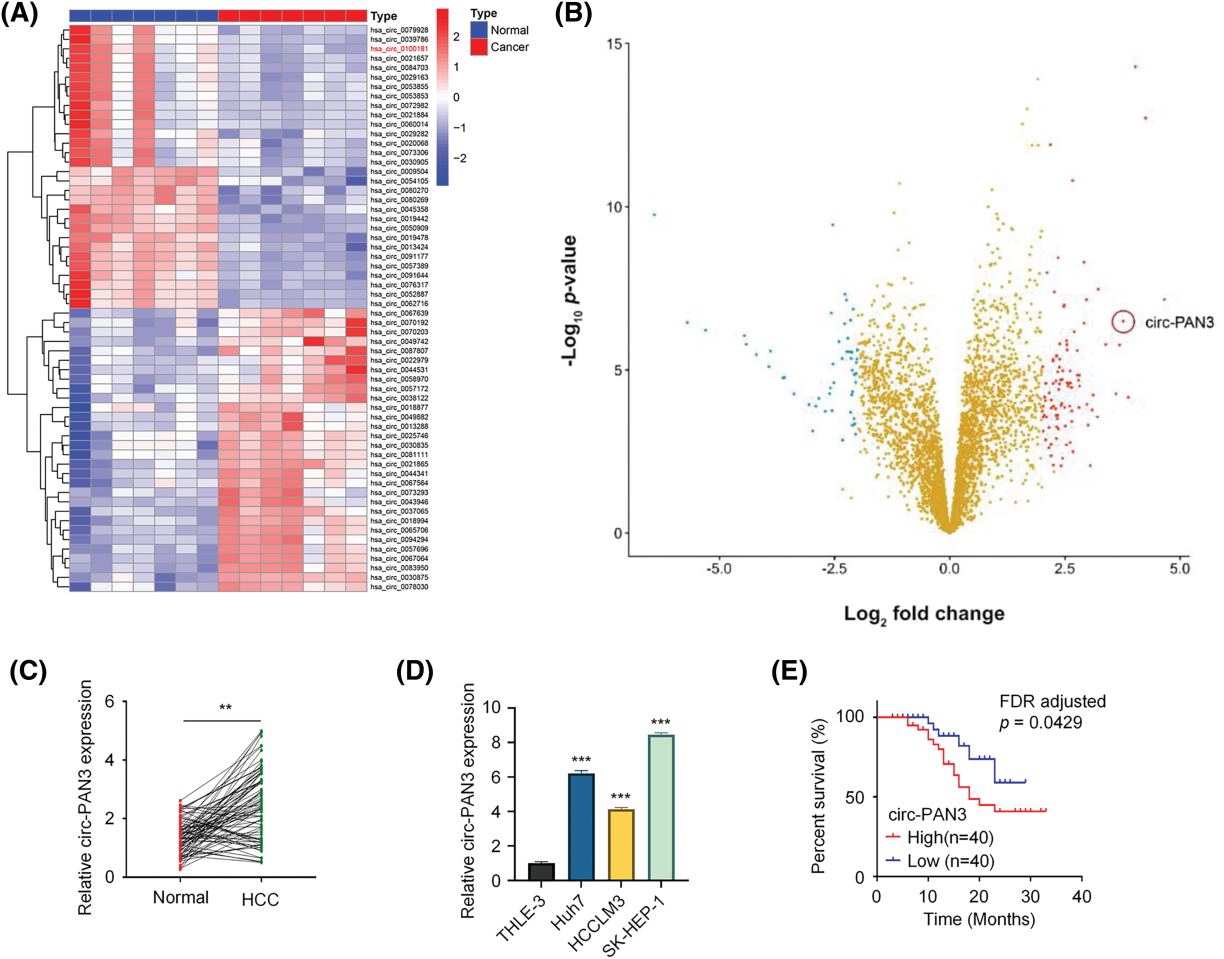
Circ-PAN3 expression in HCC tissues and cell lines and its correlation with the clinical outcomes. (A) Hierarchical clustering of top 20 upregulated and downregulated circRNAs in HCC tissues and normal tissues. (B) Volcano plots showing differential expressed genes between HCC tissues and normal tissues. (C) Circ-PAN3 expression levels in HCC tissues (*n* = 80) and adjacent normal tissues (*n* = 80) were measured using RT-qPCR. (D) Circ-PAN3 expression levels in HCC cell lines (Huh7, HCCLM3, SK-HEP-1) and human normal liver cell (THLE-3). (E) Kaplan-Meier curve analysis of the overall survival in HCC patients with high (*n* = 40) and low circ-PAN3 expression levels (*n* = 40). Data are presented as mean ± SEM; *n* ≥ 3. **p < 0.01; ***p < 0.001. HCC, Hepatocellular carcinoma.

To examine the global profile of circRNA expression in HCC, we conducted RNA microarray analysis on seven pairs of HCC and adjacent noncancerous tissues. A total of 3284 circRNAs were identified, and the top 20 upregulated and downregulated circRNAs were determined using hierarchical clustering (fold change > 2.0, *p* < 0.05, and FDR < 0.05; Fig. 1A). Volcano plots were generated to visualize the significantly differentially expressed circRNAs (Fig. 1B). Among the top five upregulated circRNAs identified through microarray analysis, circ-PAN3 was the only circRNA that showed upregulation in HCC cell lines compared to normal liver cell line THLE-3 (Fig. S1). To validate the upregulation of circ-PAN3 in HCC, we quantified circ-PAN3 transcripts in 80 pairs of HCC tumor samples and matched adjacent normal tissues. Consistent with the microarray data, circ-PAN3 expression was significantly higher in tumor samples compared to that of the normal tissues (Fig. 1C; *p* < 0.001). Furthermore, we measured circ-PAN3 expression levels in HCC cell lines (Huh7, HCCLM3, SK-HEP-1) and normal liver cell line THLE-3. Similarly, circ-PAN3 expression levels were significantly elevated in HCC cell lines compared to normal liver cells (Fig. 1D; *p* < 0.001).

Moreover, we analyzed the correlations between circ-PAN3 expression levels and different clinical characteristics in HCC patients. It was found that circ-PAN3 expression levels were significantly correlated with tumor size, TNM staging, and lymph node metastasis. However, there was no significant correlation between circ-PAN3 expression levels and age, sex, or tumor differentiation (Table 1). Additionally, patients with elevated circ-PAN3 levels suffered from an overall poorer survival (Fig. 1E; *p* = 0.0346, FDR corrected *p* = 0.0429). Overall, the high expression of circ-PAN3 in HCC tissues and cell lines, along with its positive correlation with poor patient survival, strongly suggests that elevated circ-PAN3 expression is implicated in HCC progression.

**Table 1:** Correlation analysis between circ-PAN3 expression levels and the clinicopathological features in patients with hepatocellular carcinoma

Variable		circ-PAN3		*p* value
		Low	High	
Age(years)				
	≤50	19	23	0.502
	>50	21	17	
Sex				
	Female	16	11	0.344
	Male	24	29	
Tumor size				
	≤5	24	11	0.007
	>5	16	29	
TNM				
	I/II	21	10	0.021
	III/IV	19	30	
Lymph node metastasis				
	Yes	11	26	0.002
	No	29	14	
Tumor differentiation				
	High	8	16	0.086
	Middle	13	11	
	Low	19	11	

### Knockdown of circ-PAN3 inhibits HCC cell proliferation and tumor growth

**Figure 2:**
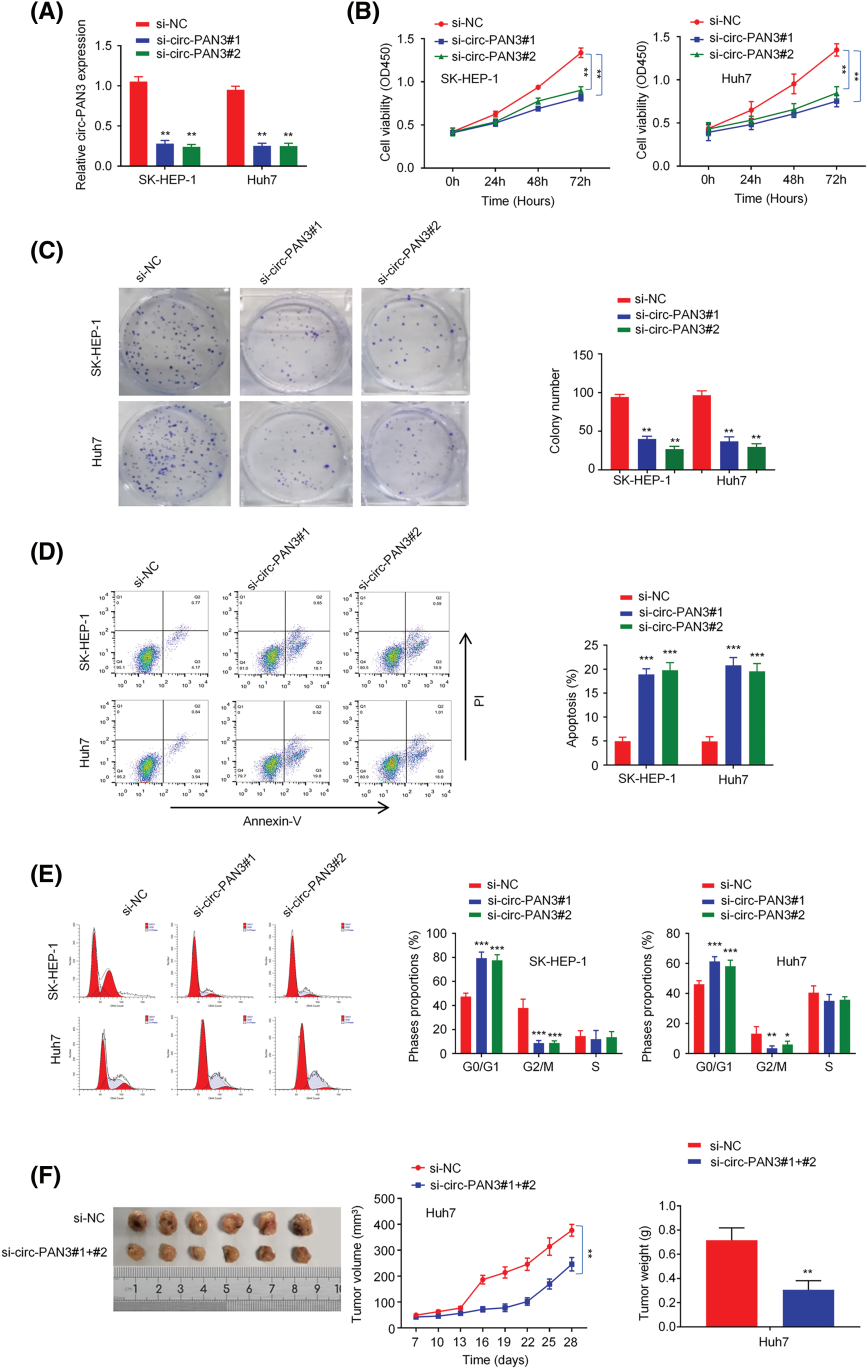
Effect of circ-PAN3 on the cell growth and tumor formation of HCC cells. (A) circ-PAN3 expression detection in SK-Hep-1 and Huh7 cells after the transfection of control and circ-PAN3 siRNAs. (B) Cell growth analysis in SK-Hep-1 (left) and Huh7 (right) cells upon circ-PAN3 siRNA transfection. (C) Colony formation assays in SK-Hep-1 (left) and Huh7 (right) cells upon circ-PAN3 siRNAs transfection (left), and cell colony numbers were quantified (right). (D) Cell apoptosis was detected using flow cytometry (left), and the percentage of apoptotic cells was quantified (right) in SK-Hep-1 (left) and Huh7 (right) cells upon circ-PAN3 siRNA transfection. (E) Quantification of the cell cycle distribution in SK-Hep-1 (left) and Huh7 (right) cells upon circ-PAN3 siRNA transfection. (F) Huh7 cells were injected subcutaneously into the flanks of nude mice. The mice were administrated with si-NC or circ-PAN3 siRNAs every week (*n* = 6 animals in each group). Tumor volume was monitored every three days and the tumor mass was measured after 4 weeks. Data are presented as mean ± SEM; *n* ≥ 3. *p < 0.05; **p < 0.01; *** *p* < 0.001.

If circ-PAN3 were a genuine promoter of HCC progression, knocking down circ-PAN3 would be expected to suppress cell proliferation. To investigate this, we conducted experiments to knock down circ-PAN3 expression in two HCC cell lines, SK-Hep-1 and Huh7, which exhibited high levels of circ-PAN3 (Fig. 1E). Following transfection with si-circ-PAN3, the expression levels of circ-PAN3 were significantly reduced (Fig. 2A). Subsequently, we assessed the phenotype of circ-PAN3 knockdown through cell viability, colony formation, and cell proliferation assays. Both cell lines transfected with si-circ-PAN3 exhibited significantly inhibited cell proliferation and colony formation (Figs. 2B and 2C). Additionally, analysis of cell apoptosis revealed an increased apoptosis rate in HCC cells with circ-PAN3 knockdown compared to the control groups (Fig. 2D). Furthermore, cell cycle analysis demonstrated that circ-PAN3 knockdown led to an increased percentage of cells in the G0/G1 phase and a decreased rate in the G2/M phase (Fig. 2E). These findings collectively suggest that circ-PAN3 knockdown induces cell cycle arrest and cell apoptosis, resulting in impaired HCC cell growth. To further validate the effect of circ-PAN3 knockdown on HCC tumor formation, we established an HCC tumor xenograft model in nude mice by injecting Huh7 cells, followed by the administration of si-NC and si-circ-PAN3. *In vivo* silencing of circ-PAN3 significantly curtailed the tumor formation of Huh7 cells (Fig. 2F). The largest diameter and volume of the tumors were measured as 0.95 cm and 385 mm^3^ in the control group, while the tumor sizes were reduced by nearly 50% at the end of the experiment.

### Circ-PAN3 is positively correlated with cyclin D1 expression in HCC cells

**Figure 3:**
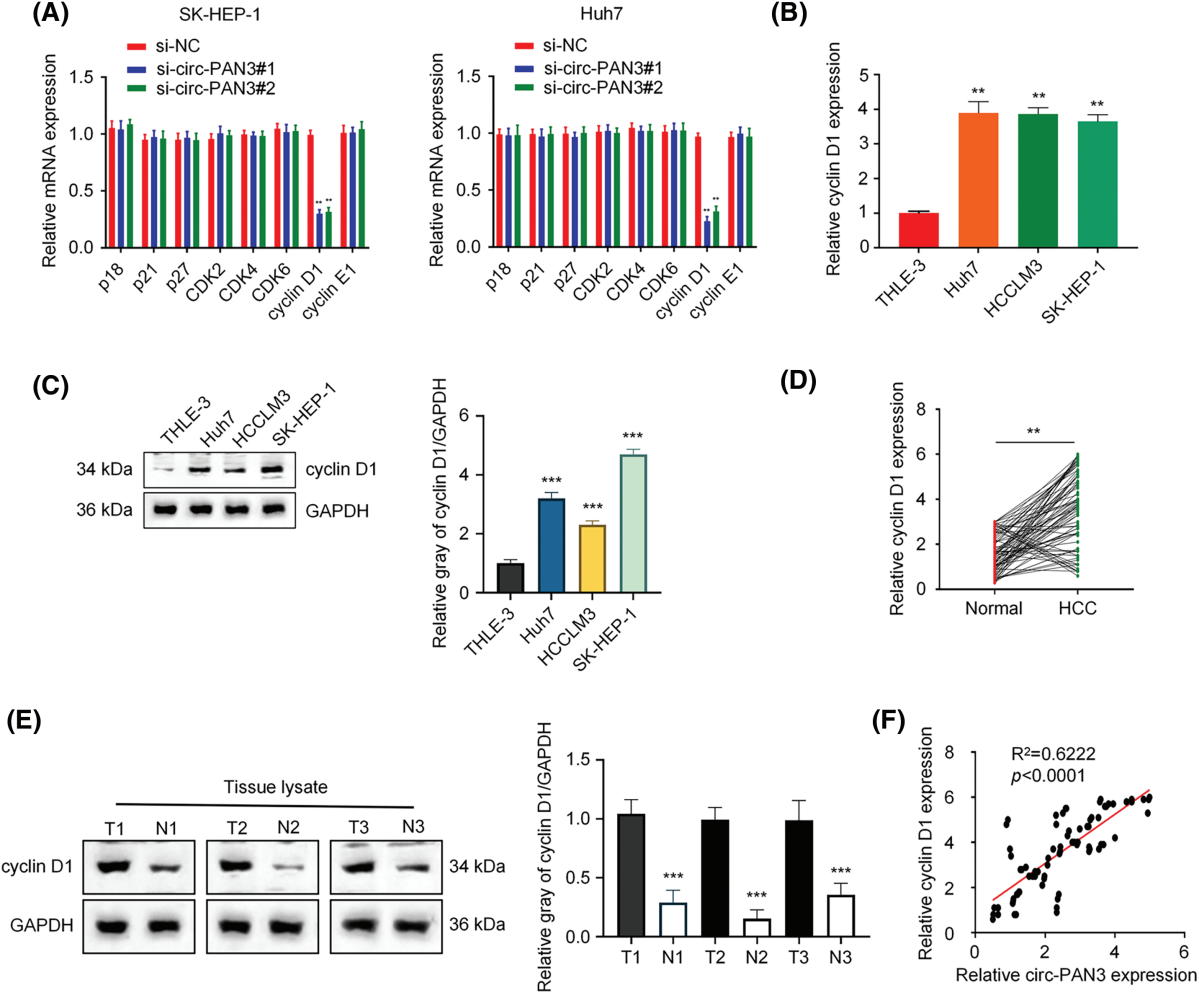
Circ-PAN3 promotes cyclin D1 expression in HCC cells. (A) The expression levels of cell cycle-associated genes in SK-Hep-1 (left) and Huh7 (right) cells transfected with circ-PAN3 siRNAs. (B) Cyclin D1 expression levels in HCC cell lines (Huh7, HCCLM3, SK-HEP-1) and human normal liver cell (THLE-3) were measured using RT-qPCR. (C) Cyclin D1expression levels in HCC cell lines (Huh7, HCCLM3, SK-HEP-1) and human normal liver cell (THLE-3) were examined by Western blot analysis (left) and the protein band intensities were quantified (right). (D) Cyclin D1 expression levels in HCC tumor tissues (*n* = 80) and adjacent normal tissues (*n* = 80) were measured using RT-qPCR (left) and (E) the protein levels were detected by Western blotting (3 pairs of samples). (F) The correlation between the expression levels of circ-PAN3 and cyclin D1 in HCC tumor tissues was analyzed using Pearson’s correlation analysis. Data are presented as mean ± SEM; *n* ≥ 3. **p < 0.01; ***p < 0.001.

Since cell cycle arrest was induced in HCC cell lines by circ-PAN3 knockdown, we hypothesized that circ-PAN3 could influence the expression or activity of cell cycle-associated genes. Our findings revealed that knockdown of circ-PAN3 led to a decrease in cyclin D1 expression, while the expression of other cell cycle-associated genes remained unaffected in SK-Hep-1 and Huh7 cells (Fig. 3A). Furthermore, HCC cell lines with higher levels of circ-PAN3 exhibited elevated levels of cyclin D1 expression (Figs. 3B and 3C). Additionally, both mRNA and protein levels of cyclin D1 were significantly higher in HCC tumor tissues compared to adjacent normal tissues (Figs. 3D and 3E). Pearson’s correlation analysis demonstrated a positive correlation between the expression levels of circ-PAN3 and cyclin D1 (Fig. 3F). These results indicate a positive association between circ-PAN3 expression and cyclin D1 expression in HCC, suggesting a functional interplay of circ-PAN3 on cyclin D1.

### Circ-PAN3 sponges miR-153 to regulate cyclin D1 in HCC cells

**Figure 4:**
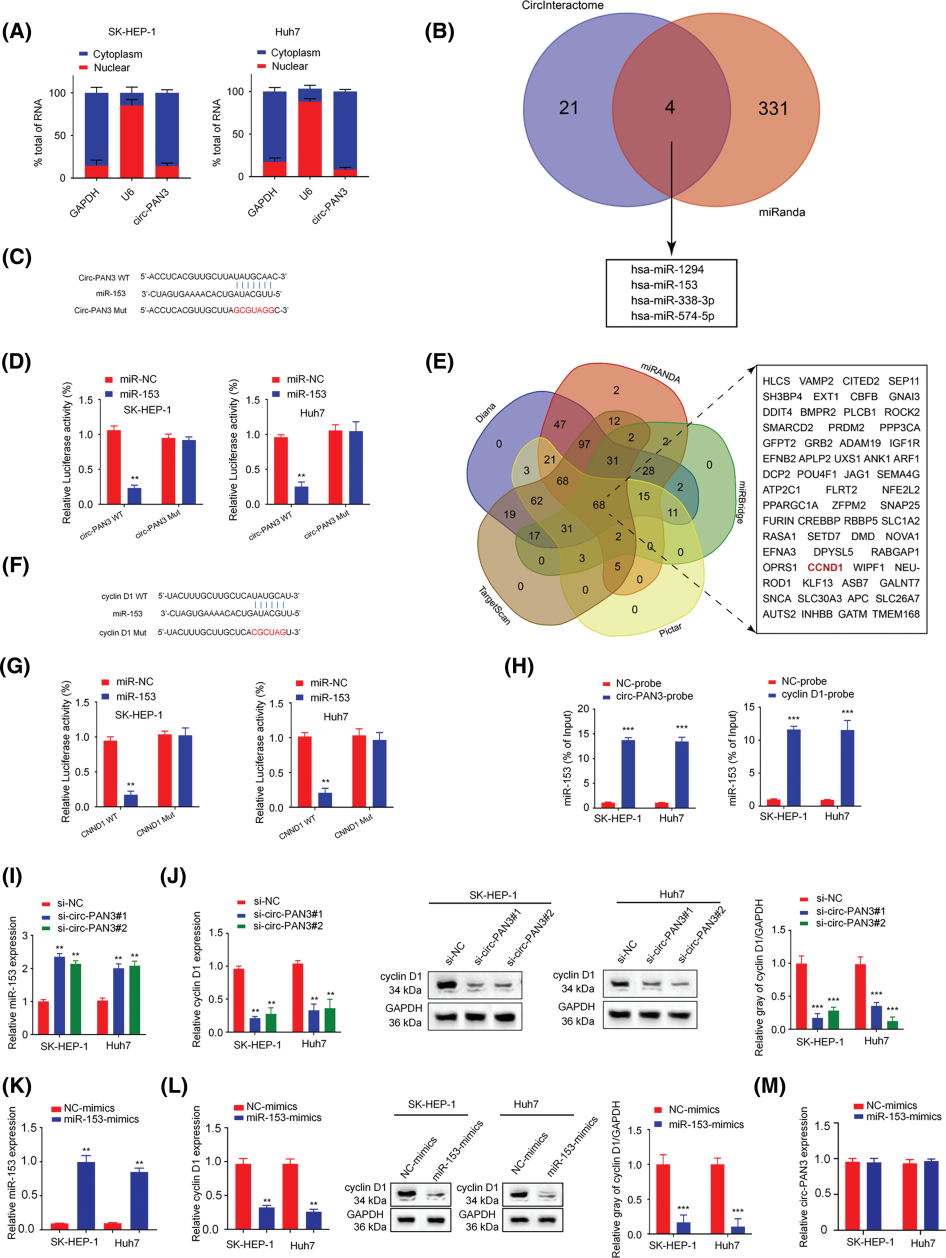
Circ-PAN3 sponges miR-153 to regulate cyclin D1 expression in HCC cells. (A) RT-qPCR analysis of circ-PAN3 transcripts in the cytoplasmic or nuclear fractions of SK-HEP-1 (left) and Huh7 (right) cells. (B) Venn diagram showing the predicted miRNA targets of circ-PAN3. (C) The potential binding region and the mutated sequences between circ-PAN3 and miR-153. (D) miR-153 was validated as a target of circ-PAN3 using dual-luciferase reporter assays in SK-HEP-1 (left) and Huh7 (right) cells. (E) Venn diagram showing the predicted mRNA targets of miR-153. (F) The potential binding region and and the mutated sequences between miR-153 and CCND1. (G) Cyclin D1 was validated as a direct target of miR-153 using dual-luciferase reporter assays in SK-HEP-1 (left) and Huh7 (right) cells. (H) RNA pull-down analysis of miR-153 interaction using biotinylated-circ-PAN3 probe and control probe in SK-HEP-1 and Huh7 cells (left). RNA pull-down analysis of miR-153 interaction with the biotinylated-Cyclin D1 probe or control probe in SK-HEP-1 and Huh7 cells (right). (I) The expression levels of miR-153 in SK-HEP-1 and Huh7 cells transfected with circ-PAN3 siRNAs were detected by RT-qPCR. (J) The expression levels of cyclin D1 in SK-HEP-1 and Huh7 cells transfected with circ-PAN3 siRNAs were detected by RT-qPCR (left) and Western blotting (right). (K) The expression levels of miR-153 in SK-HEP-1 and Huh7 cells transfected with miR-153 mimics were detected by RT-qPCR. (L) The expression levels of cyclin D1 in cells transfected with miR-153 mimic were detected by RT-qPCR (left) and Western blotting (right). (M) The expression levels of circ-PAN3 in cells transfected with miR-153 mimic in SK-HEP-1 and Huh7 cells were detected by RT-qPCR. Data are presented as mean ± SEM; *n* ≥ 3. **p < 0.01; ***p < 0.001; ^##^*p* < 0.01.

To determine the subcellular localization of circ-PAN3, we analyzed subcellular RNA levels in the cytoplasmic and nuclear fractions of SK-Hep-1 and Huh7 cells. The results showed that circ-PAN3 was primarily concentrated in the cytoplasm (Fig. 4A). Next, CircInteractome and miRanda online resources were utilized to identify potential interactors of circ-PAN3 and Cyclin D1, respectively. The prediction revealed that miR-153, miR-1294, miR-338-3p, and miR-574-5p could bind to both circ-PAN3 and Cyclin D1 (Fig. 4B). To confirm the interaction between miR-153 and circ-PAN3, we examined the expression of miR-1294, miR-153, miR-338-3p, and miR-574-5p after transfecting HCC cell with circ-PAN3 expression vector. We found that only miR-153 was downregulated upon circ-PAN3 expression (Fig. S2). Subsequently, we performed a dual luciferase assay to further investigate the interaction between miR-153 and circ-PAN3. The results showed that miR-153 mimic transfection significantly reduced luciferase activity of circ-PAN3-WT vector, while no such inhibition was detected in the MUT reporter (Figs. 4C and 4D). These data suggest the interaction between circ-PAN3 and miR-153.

Additionally, we predicted potential targets of miR-153 using Diana, TargetScan, miRanda, miRBridge, and Pictar online databases. The analysis identified 68 downstream targets that were overlapped across the five datasets (Fig. 4E). Among these targets, CCND1 was reported as a cell cycle-associated gene. The results of luciferase assays showed that miR-153 mimic significantly reduced the luciferase activity in SK-Hep-1 and Huh7 cells transfected with CCND1-WT vector, and the activity of CCND1-MUT vector was not affected (Figs. 4F and 4G). Moreover, RNA pull-down experiment with the biotinylated-circ-PAN3 probe confirmed the physical association between circ-PAN3 and miR-153 in SK-Hep-1 and Huh7 cells (Fig. 4H). RNA pull-down using biotinylated-cyclin D1 probe also supported the direct interaction between CNND1 and miR-153 in SK-Hep-1 and Huh7 cells (Fig. 4H).

The miR-153 expression level was measured after knockdown of circ-PAN3. It was found that circ-PAN3 knockdown significantly increased miR-153 expression level (Fig. 4I). Additionally, the expression level of cyclin D1 was quantified when circ-PAN3 expression was knocked down. Circ-PAN3 knockdown reduced cyclin D1 expression level in SK-Hep-1 and Huh7 cells at both RNA and protein levels (Figs. 4I and 4J). We further investigated whether miR-153 could regulate the expression level of cyclin D1 and found that miR-153 mimic decreased both RNA and protein levels of cyclin D1 (Figs. 4K and 4L). However, miR-153 overexpression did not impinge on circ-PAN3 expression level (Fig. 4M). These findings collectively suggest that circ-PAN3 is an upstream regulator which serves as a molecular sponge for miR-153, and miR-153 functions a s negative regulator for cyclin D1.

### Inhibiting miR-153 or overexpressing cyclin D1 rescues cell proliferation suppression induced by circ-PAN3 knockdown

**Figure 5:**
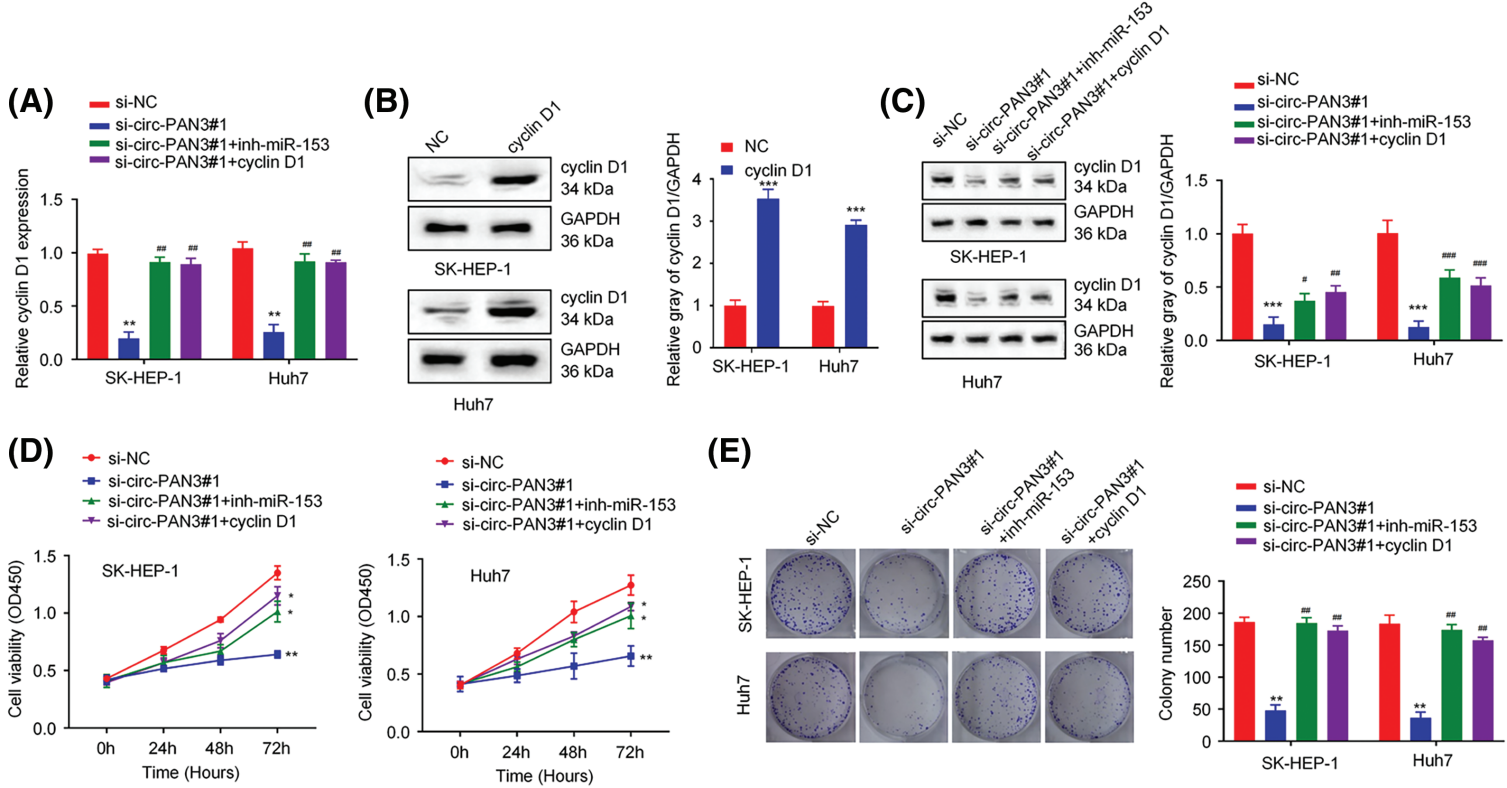
Circ-PAN3/miR153/cyclin D1 axis regulates cell growth in HCC cells. (A) The expression of cyclin D1 was measured by RT-qPCR in SK-HEP-1 and Huh7 cells transfected with si-NC, circ-PAN3-siRNA, circ-PAN3-siRNA plus miR-153 inhibitor, or circ-PAN3-siRNA and cyclin D1 expression plasmid. (B) The expression of cyclin D1 was measured in SK-HEP-1 and Huh7 cells transfected with empty vector (NC) or cyclin D1 expression plasmid. (C) The expression of cyclin D1 was measured (left) and quantified (right) by Western blotting in SK-HEP-1 and Huh7 cells, which were transfected with si-NC, circ-PAN3-siRNA, circ-PAN3-siRNA plus miR-153 inhibitor, or circ-PAN3-siRNA and cyclin D1 expression plasmid. (D) Cell proliferation analysis in SK-HEP-1 (left) and Huh7 (right) cells transfected with si-NC, circ-PAN3-siRNA, circ-PAN3-siRNA plus miR-153 inhibitor, or circ-PAN3-siRNA and cyclin D1 expression plasmid. (E) Colony formation assays in SK-HEP-1 (left) and Huh7 (right) cells transfected with si-NC, circ-PAN3-siRNA, circ-PAN3-siRNA and miR-153 inhibitor, or circ-PAN3-siRNA and cyclin D1 expression plasmid. Data are presented as mean ± SEM; *n* ≥ 3. *p < 0.05; **p < 0.01; ***p <0.001; compared with si-NC group; and ^#^*p* < 0.05; ^##^*p* < 0.01; ^###^*p* < 0.001 compared with si-circ-PAN3#1 group.

To further clarify the relationship between circ-PAN3, miR-153, and cyclin D1, rescue experiment was conducted in HCC cells by transfecting miR-153 inhibitor or cyclin D1 expression vector after circ-PAN3 knockdown. The results demonstrated that the suppression of cyclin D1 expression caused by circ-PAN3 knockdown was reversed when miR-153 inhibitor or cyclin D1 overexpression was introduced (Figs. 5A–5C). Moreover, the impaired cell proliferation and colony formation observed in circ-PAN3 knockdown cells were restored upon the addition of miR-153 inhibitor or cyclin D1 overexpression (Figs. 5D and 5E). Overall, these findings provide further support for the involvement of the circ-PAN3/miR-153/cyclin D1 axis in modulating HCC cell growth.

## Discussion

CircRNAs play a significant role in the development and advancement of various types of tumors. This study revealed that circ-PAN3 expression is notably increased in HCC tissues and cell lines, and it is associated with a poor survival rate. Additionally, it was discovered that circ-PAN3 functions as a competitive endogenous RNA to suppress the activity of miR-153. The reduction of miR-153 level subsequently causes an upregulation of cyclin D1, ultimately augmenting the proliferation of HCC cells. Furthermore, the inhibition of HCC cell proliferation caused by circ-PAN3 knockdown can be reversed by overexpressing cyclin D1. Thus, a novel molecular module, circ-PAN3/miR-153/cyclin D1, has been identified as a crucial regulatory mechanism for the progression of HCC.

Circ-PAN3 is a newly identified circRNA located on 13q12.2 [15]. Its deregulation has been associated with various diseases [15,16]. In acute myeloid leukemia, circ-PAN3 promotes drug resistance by inducing autophagy through modulating AMPK/mTOR pathway [15]. Additionally, circ-PAN3 acts as a critical mediator of chemoresistance in leukemia cells through targeting miR-153-5p and miR-183-5p-XIAP regulatory axis [15]. In cardiac fibrosis, circ-PAN3 exhibits profibrotic effects via the miR-221/FoxO3/ATG7 axis [21]. Our study suggests circ-PAN3 as a tumor-promoting factor in HCC progression. Silencing circ-PAN3 could be developed into an intervention strategy in the clinical management of HCC patients. Although circRNAs are widely implicated in tumorigenesis, the molecular mechanism has not been fully understood. Our study provides further insights into the mechanism by which circ-PAN3 modulates HCC cell growth through regulating cell cycle regulator.

Our data revealed a downregulation of cyclin D1 expression upon circ-PAN3 knockdown. Cyclin D1 is known to play a pro-tumorigenic role in HCC and is crucial for cell proliferation and invasion [22,23]. The regulation of cyclin D1 expression involves various mechanisms, such as autophagy degradation and miRNA modulation [24]. Recent studies have also shed light on the role of cyclin D1 in cell apoptosis. For instance, Wu et al. demonstrated that blocking cyclin D1 using a specific antibody induced G1 phase arrest and triggered apoptotic cell death by reducing the phosphorylation level of retinoblastoma protein (Rb) in HCC [25]. Similarly, Wang et al. showed that miR-193a-3p inhibited the propagation of HCC cells and promoted apoptosis by inhibiting cyclin D1 [26]. Moreover, Huang et al. found that isoliquiritigenin suppressed cyclin D1 expression, resulting in cell cycle arrest and apoptosis in HCC [27]. Chen et al. provided evidence that cyclin D1 knockdown attenuated cell proliferation, decreased cell percentage in S and G2/M phase, induced apoptosis and suppressed the tumorigenicity of HCC cells [27]. Cyclin D1 expression can be positively regulated by X-linked inhibitor of apoptosis protein (XIAP) through E3 ligase-mediated phosphorylation [19]. Notably, circ-PAN3 has been shown to positively regulate XIAP, leading to chemoresistance in acute myeloid leukemia [19]. We observed cyclin D1 expression is positively regulated by circ-PAN3 and negatively regulated by miR-153 in HCC cells. Whether this molecular axis is also implicated in the development of other cancer types warrants further investigation.

CircRNAs have been found to exert their functional regulation through various mechanisms, such as genomic targeting, *cis* or *trans* regulations, and antisense interference [6]. In recent years, there has been significant interest in the role of circRNA as competitive endogenous RNA in different types of cancer [11,13]. Competitive endogenous RNA can disrupt the activities of target miRNAs, leading to an upregulation in the expression of miRNA-targeted genes [28]. In the current study, we demonstrated that miR-153 could bind to both circ-PAN3 and cyclin D1. Previous research has shown that LINC00858 and lncRNA CDKN2BAS can act as sponges for miR-153, thereby modulating the expression of downstream targets in HCC [29,30]. Our findings suggest circ-PAN3 as a molecular sponge for miR-153, and circ-PAN3 upregulation in HCC facilitates cyclin D1 overexpression. miR-153 has been reported to inhibit the proliferation and invasion of cancer cells through various targets, including Rabl3, ARHGAP18, SNAI1, and Wnt/β-catenin [29–32]. Our study provides novel evidence that cyclin D1 is another target of miR-153 in HCC, suggesting multiple mechanisms involved in HCC tumorigenesis and progression.

## Conclusion

To sum up, our study demonstrated that circ-PAN3 acts as a sponge for miR-153, resulting in the upregulation of cyclin D1 which ultimately contributes to the progression of HCC. These findings suggest that circ-PAN3 plays a crucial role in HCC progression and could potentially serve as a novel prognostic and therapeutic target for HCC diagnosis and treatment.

## Supplementary Materials

Figure S1 The expressions of other circRNA candidates identified by microarray were examined by RT-qPCR in HCC cell lines (Huh7, HCCLM3, SK-HEP-1) and human normal liver cell (THLE-3). ^##^p < 0.01.

Figure S2 The expression levels of miRNA candidates were analyzed by RT-qPCR in SK-HEP-1 cells transfected with circ-PAN3 siRNA or control siRNA. **p <0.01; ^##^p < 0.01.

## Data Availability

The datasets generated and/or analyzed during the current study are not publicly available due to the need of permission from the IRB, but are available from the corresponding author on reasonable request (ryqin@tjh.tjmu.edu.cn).

## Supplementary Materials

The supplementary material is available online at https://doi.org/10.32604/or.2024.046774.
