# Supplementary figures and images for: Circ-PAN3 facilitates hepatocellular carcinoma growth via sponging miR-153 and upregulating cyclin D1
Source: Oncol Res. 2025 Jan 16;33(2):369–80. doi: 10.32604/or.2024.046774 (PMC11753995; doi:10.32604/or.2024.046774)

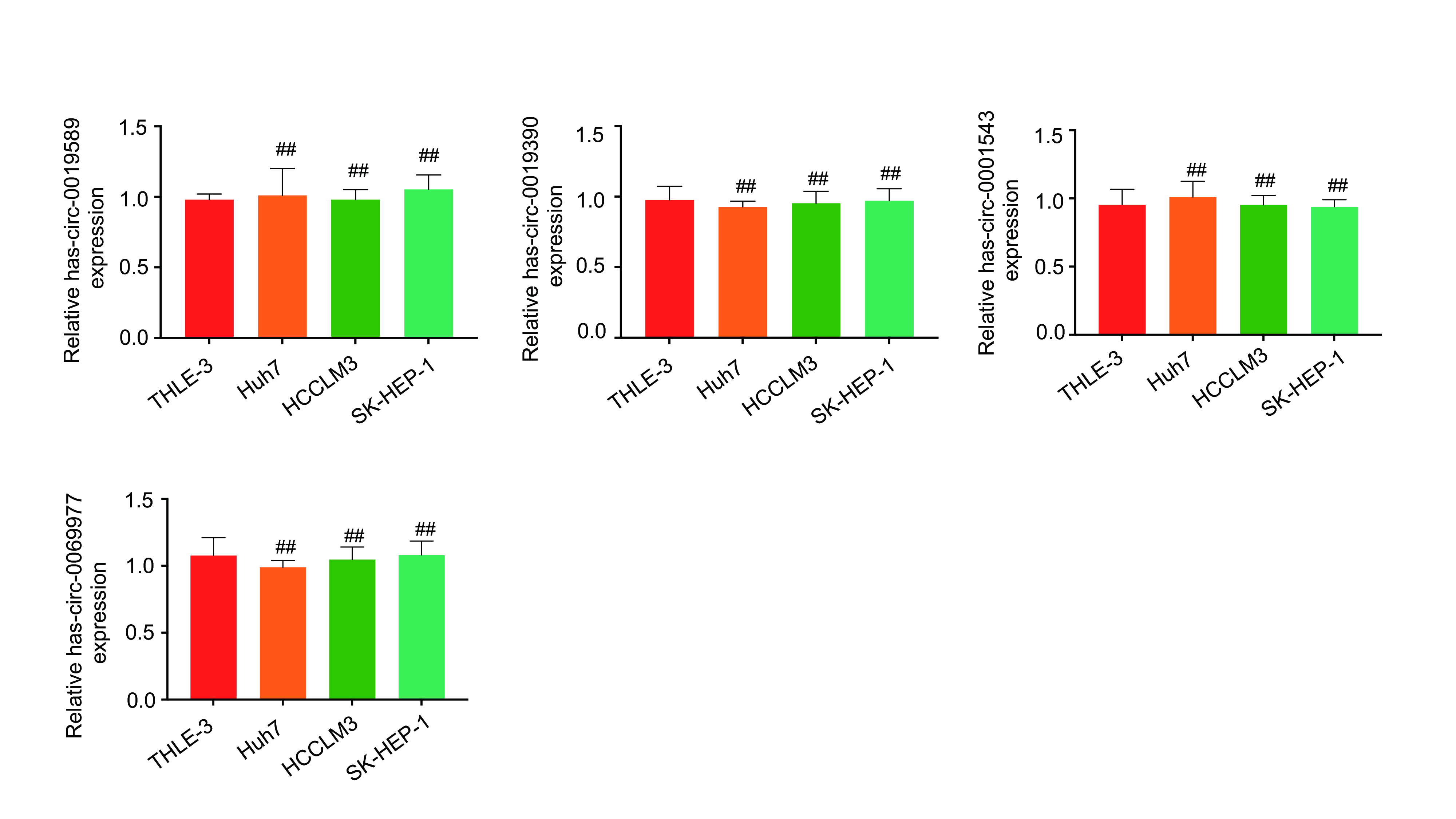

Supplement: Figure S1 [file OncolRes-33-46774-s001.tif]

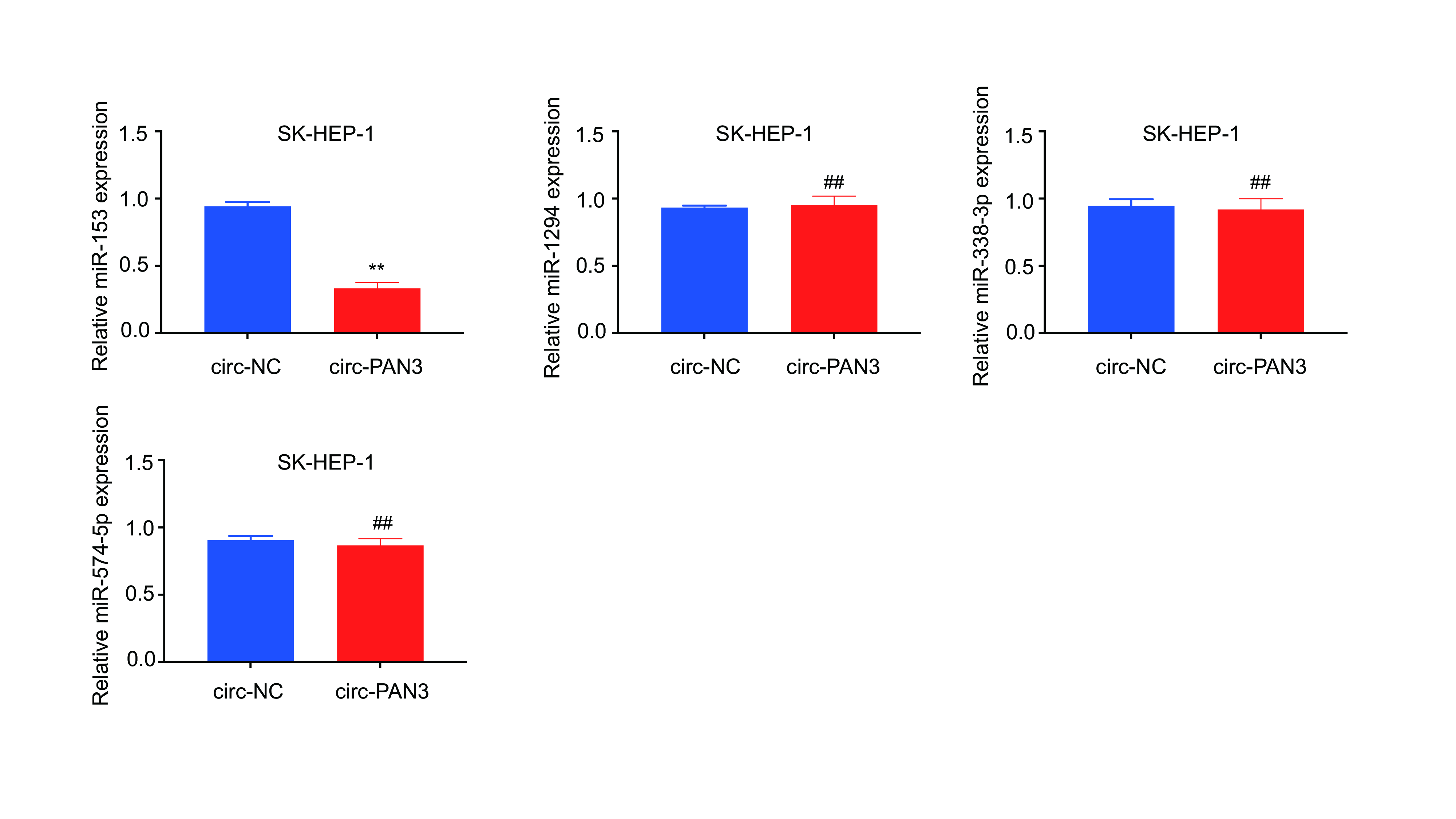

Supplement: Figure S2 [file OncolRes-33-46774-s002.tif]
